# *Brucella* and Osteoarticular Cell Activation: Partners in Crime

**DOI:** 10.3389/fmicb.2017.00256

**Published:** 2017-02-20

**Authors:** Guillermo H. Giambartolomei, Paula C. Arriola Benitez, M. Victoria Delpino

**Affiliations:** Instituto de Inmunología, Genética y Metabolismo – Consejo Nacional de Investigaciones Cientificas y Tecnicas – Universidad de Buenos AiresBuenos Aires, Argentina

**Keywords:** osteoarticular brucellosis, B and T cells and *Brucella*, synoviocyte, osteoblast, osteoclastogenesis

## Abstract

Osteoarticular brucellosis is the most common presentation of human active disease although its prevalence varies widely. The three most common forms of osteoarticular involvement are sacroiliitis, spondylitis, and peripheral arthritis. The molecular mechanisms implicated in bone damage have been recently elucidated. *B. abortus* induces bone damage through diverse mechanisms in which TNF-α and the receptor activator of nuclear factor kappa-B ligand (RANKL)-the natural modulator of bone homeostasis are involved. These processes are driven by inflammatory cells, like monocytes/macrophages, neutrophils, Th17 CD4^+^ T, and B cells. In addition, *Brucella abortus* has a direct effect on osteoarticular cells and tilts homeostatic bone remodeling. These bacteria inhibit bone matrix deposition by osteoblasts (the only bone cells involved in bone deposition), and modify the phenotype of these cells to produce matrix metalloproteinases (MMPs) and cytokine secretion, contributing to bone matrix degradation. *B. abortus* also affects osteoclasts (cells naturally involved in bone resorption) by inducing an increase in osteoclastogenesis and osteoclast activation; thus, increasing mineral and organic bone matrix resorption, contributing to bone damage. Given that the pathology induced by *Brucella* species involved joint tissue, experiments conducted on synoviocytes revealed that besides inducing the activation of these cells to secrete chemokines, proinflammatory cytokines and MMPS, the infection also inhibits synoviocyte apoptosis. *Brucella* is an intracellular bacterium that replicates preferentially in the endoplasmic reticulum of macrophages. The analysis of *B. abortus*-infected synoviocytes indicated that bacteria also replicate in their reticulum suggesting that they could use this cell type for intracellular replication during the osteoarticular localization of the disease. Finally, the molecular mechanisms of osteoarticular brucellosis discovered recently shed light on how the interaction between *B. abortus* and immune and osteoarticular cells may play an important role in producing damage in joint and bone.

## Osteoarticular Brucellosis- Clinical Features

Osteoarticular brucellosis is well documented as a major public health problem in several countries, particularly in the Middle East, the Mediterranean region and Central and South America (Pappas et al., 2006). Although osteoarticular involvement is the most common focal complication of brucellosis; clinical manifestations also include neurological, heart and liver complications (Buzgan et al., 2010). Its prevalence varies from one report to another, but a recent study has revealed that as many as 47% of brucellosis patients experienced osteoarticular complications (Turan et al., 2011). It can take place at early stages of disease, at any time during the course of illness or some features can be present at the onset of the disease. There are three clinical forms: peripheral arthritis, sacroiliitis, and spondylitis. Peripheral arthritis is the most common one and it affects knees, hips, ankles, and wrists in the context of acute infection (Pappas et al., 2005). The clinical features include joint pain, which may be severe, joint swelling, tenderness; increased local warmth and limitation of movement (Gotuzzo et al., 1982; Rajapakse, 1995; Al-Eissa, 1999; Shaalan et al., 2002). Sacroiliitis also occurs in the context of acute brucellosis. The third form of the osteoarticular disease, spondylitis, remains extremely difficult to treat and often seems to result in residual bone damage (Pappas et al., 2005). The lumbar spine is the usual site of involvement.

The mechanisms involved in bone damage due to *Brucella* infection are not fully understood. Bone damage can be attributed to the direct action of the bacteria or to an immunopathological process due to inflammation triggered by innate immunity. No secreted proteases, toxins or lytic enzymes have been described so far in the bacteria; therefore, it is unlikely that this fact causes a direct deleterious effect, pointing out to innate immune responses as the major cause of osteoarticular pathology.

## Interplay Between Bone and Immune System

The skeleton allows locomotion, calcium storage, and harboring hematopoietic stem cells from which blood and immune cells are derived. Although bone appears to be metabolically inert, it is actually a dynamic organ. Bone is composed of cells and an extracellular matrix which becomes mineralized by the deposition of calcium hydroxyapatite, which gives bone rigidity and strength. Bone has three different cell types: osteoblasts -or bone-forming cells-, osteoclasts -or bone-resorbing cells, whose functions are intimately linked (Ikeda and Takeshita, 2016) and osteocytes, which are terminally differentiated osteoblasts embedded within the mineralized bone matrix. Bone remodeling, a process coordinated between formation and degradation of bone managed by osteoblasts and osteoclasts, respectively, ensures bone homeostasis in healthy individuals. In order to balance bone formation and resorption, osteoblasts secrete RANKL that regulate the differentiation of osteoclasts, and osteocytes are the source of the Wnt antagonist sclerostin, and the Wnt signaling regulated by sclerostin regulate the activity of osteoblast (Karner et al., 2016), and osteocytes also secrete RANKL that regulate osteoclasts activity (Chen et al., 2015).

Several years of investigation have highlighted the interactions between bone and immune cells as well as their overlapping regulatory mechanisms (El-Jawhari et al., 2016). For instance, osteoclasts originate from the same myeloid precursor cells that give rise to macrophages and myeloid dendritic cells. On the other hand, osteoblasts regulate hematopoietic stem cell niches from which blood and immune cells are derived. Moreover, many of the soluble mediators of immune cells, including cytokines and growth factors, regulate the activities of osteoblasts and osteoclasts.

In physiological conditions, “canonical” osteoclast formation requires macrophage colony-stimulating factor (M-CSF) and receptor activator factor of nuclear factor kB ligand (RANKL) (Lampiasi et al., 2016). These cytokines act on cells of the monocyte-macrophage lineage, inducing their fusion to form multinucleated active resorbing cells. In the bone milieu, M-CSF is produced by osteoblasts and bone marrow stromal cells. It induces proliferation of osteoclast precursors, and differentiation and survival of mature osteoclasts (Fuller et al., 1993; Tanaka et al., 1993). M-CSF induces RANKL receptor expression, RANK, on mononuclear osteoclast precursors which then interacts with membrane-bound RANKL on surrounding osteoblasts and stromal cells to initiate osteoclasts differentiation (Yasuda et al., 1998). RANKL is present as both a transmembrane molecule and a secreted form. Its interaction with RANK is opposed by osteoprotegerin (OPG), a neutralizing soluble decoy receptor produced by marrow stromal cells and osteoblasts (Grundt et al., 2009). In addition to M-CSF and RANKL, a number of other cytokines and growth factors are known to substitute these two molecules and induce a “non-canonical” osteoclast formation (Lampiasi et al., 2016). Bone-marrow-derived and circulating osteoclast precursors are capable of differentiating into osteoclasts in the presence of M-CSF and substitutes for RANKL such as TNF-α, LIGHT (a receptor expressed in T lymphocytes), APRIL (a proliferation inducing ligand), BAFF (a B cell activating factor), the nerve growth factor, insulin-like growth factor (IGF)-I and II, TGF-β, IL-6, IL-11, IL-8; or in the presence of RANKL and substitutes for M-CSF such as a vascular endothelial growth factor, placental growth factor, FLt-3 ligand and hepatocyte growth factor (Lampiasi et al., 2016). Interestingly, IL-1, IL-7, IL-17, and IL- 23 have also been involved in non-canonical osteoclastogenesis (Mori et al., 2013), mostly by inducing indirectly osteoclastogenesis and promoting RANKL release from other cells (Ikeda and Takeshita, 2016) and RANK on osteoclast precursors (Adamopoulos et al., 2010). However, it has been demonstrated that Th17 cells produce RANKL by themselves (Adamopoulos et al., 2010). IL-17 also enhances local inflammation and increases the production of inflammatory cytokines which further promote RANKL expression and activity (El-Jawhari et al., 2016). Most of these molecules are also involved in the immune system regulation and this may explain some of the cross-talk between immune and bone cells (D’Amelio et al., 2006). On the other hand, RANKL has also been involved in immune regulation (D’Amelio et al., 2006, 2009). The significance of non-canonical pathways in physiological bone resorption is uncertain. Yet, they may be important in the context of pathological bone resorption associated with inflammatory lesions of bone where high levels of these cytokines and growth factors are present.

Osteoblasts are specialized mesenchymal cells, responsible for the deposition of bone matrix and osteoclast regulation. Osteoblasts play a very important role in creating and maintaining skeletal architecture. They are the most important cells regulating bone remodeling balance. Osteoblasts express the parathyroid hormone receptor whose binding to the hormone can activate osteoclast activity by increasing serum calcium levels (Rodan et al., 1981). Together with pre-osteoblasts and stromal cells, osteoblasts produce two key factors acting on osteoclasts: RANKL and OPG (Yasuda et al., 1998). Osteoblasts were proposed to play a major role in haematopoiesis (Visnjic et al., 2001). In the bone marrow, hematopoietic stem cells intermingle with specialized microenvironments -known as stem-cell niches aimed at maintaining their pluripotency and self-renewal ability. Osteoblasts, on the trabecular bone surface, have emerged as a crucial component of this niche, where long-lived antibody-producing B cells are known to reside. CXCL12 and its receptor CXCR4 are involved in the colonization of bone marrow by these B cells and in their retention in the bone marrow, but the localization of CXCL12-expressing cells is not consistent with that of osteoblasts on the trabecular bone surface (Tokoyoda et al., 2004). As it is unclear whether the bone-forming capacity is related to the function required for the niche, it is necessary to carefully reconsider this function for osteoblasts. Recently, osteoclasts have also been involved in the mobilization of stem cells (Kollet et al., 2006), further supporting the intimate relationship between the immune and bone systems.

Although bone is normally resistant to infection, *Brucella* spp. have a tropism for the osteoarticular localization. In this review, we report the current understanding of the interaction between *Brucella*, resident bone cells and immune system cells.

## *Brucella* and Osteoblast

Infection of murine and human osteoblasts with *B. abortus* is a determining factor in the development of osteomyelitis in bone tissue. *B. abortus* interacts directly with osteoblasts, and replicates inside these cells. As a result of this interaction, modifications occur in the osteoblast metabolism. *B. abortus* inhibits osteoblast differentiation and function, leading to bone loss. Infection induces apoptosis of osteoblasts and also inhibits mineral and organic matrix deposition by these cells. Infection also induces RANKL expression, the main mediator of osteoclast differentiation. All these processes are activated through the p38 and ERK1/2 MAPK pathway (Scian et al., 2012). TNF-α secreted by *B. abortus*-infected macrophages also induces apoptosis and inhibits matrix deposition by osteoblasts (Scian et al., 2012). In addition, infection elicits secretion of chemokines and metalloproteinases (MMPs) (Scian et al., 2011b, 2012). Bone and joint damage can be the result of the inflammatory reaction elicited by MMP activity. Locally increased levels of MMPs have been found in arthritis associated with Lyme disease (Behera et al., 2005) and in periodontitis due to multiple bacteria (Soder et al., 2006). In these pathological processes the main sources of MMPs are inflammatory infiltrating cells (Song et al., 2013). Accordingly, *in vitro* studies have indicated that monocytes and neutrophils infected with *Brucella* secrete MMP-9 and proinflammatory cytokines (Zwerdling et al., 2009; Delpino et al., 2010; Scian et al., 2011b). Osteoblasts have also been shown to produce several MMPs, among which MMP-2 is particularly important because it degrades type I collagen present in bones and type II collagen present in cartilage (Burrage et al., 2006). *B. abortus* infection of osteoblasts elicits GM-CSF secretion which acts as the major mediator of the increase in MMP-2 production detected in culture supernatants from infected osteoblasts (Scian et al., 2011b). We have also observed an increase in the levels of MMP activity in the synovial fluid from a patient with prepatellar bursitis (Wallach et al., 2010). A common feature of patients with osteoarticular brucellosis is the presence of leukocyte infiltrates (including monocytes and neutrophils) in the synovial fluid of the joints (Gotuzzo et al., 1982; Ibero et al., 1997; Press et al., 2002; Kasim et al., 2004). Accordingly, *B. abortus* infection induces the secretion of IL-8 and MCP-1 by osteoblasts. In conclusion, *B. abortus* may modulate, directly and indirectly, osteoblast function to increase bone resorption.

## *Brucella* and Osteocytes

Osteocytes are the terminally differentiated forms of osteoblasts embedded in the mineralized bone matrix (Bonewald, 2011). For many years, the bone-bound osteocyte has been considered to be a relatively inactive cell with an unknown role in the bone tissue. Osteocytes are not only the most abundant cell populations of the bone lineage, which comprise up to 95% of bone cells in the adult skeleton, but also the central regulators of the differentiation and activity of both osteoblasts and osteoclasts during bone remodeling (Zhao et al., 2002). Osteocytes form an extensive and multi-functional syncytium throughout the bone. Their location within the matrix confers these cells the ability to sense stress throughout the bone, and to respond accordingly. Osteocytes respond to mechanical load by sending signals to osteoblasts and osteoclasts in the bone and modulating their activity. RANKL, NO and IGF-1 have been identified as such signal factors (Prideaux et al., 2016).

*In vitro* studies revealed that *B. abortus* may invade murine osteocytes inducing the secretion of MMP-2, RANKL, and proinflammatory cytokines. This inflammatory response induces bone marrow-derived monocytes to undergo osteoclastogenesis via RANKL and TNF-α (Pesce Viglietti et al., 2016).

Osteocytes are trapped within a mineralized matrix. In these conditions, cell-to-cell and cell-to-matrix communication in bone cells mediated by gap junctions and hemichannels, respectively, maintains bone homeostasis. Connexin 43 (Cx-43) is the predominant gap junction protein in bone and it facilitates the communication of cellular signals between cells that are required to maintain viability of osteocytes (Civitelli, 2008; Bivi et al., 2012). *B. abortus* infection inhibits Cx43 expression (Pesce Viglietti et al., 2016). Cx43 deletion in osteocyte cell culture results in increased apoptosis (Bivi et al., 2012). Integrins also control the fate and function of cells by influencing not only their proliferation and differentiation but also apoptosis (Streuli, 2009). *B. abortus* infection reduces the expression of Cx43 expression but does not modify integrin expression on murine osteocytes, which results in the absence of apoptosis (Pesce Viglietti et al., 2016). However, when osteocytes interact with supernatants from *B. abortus-*infected macrophages, the expression of Cx43 is inhibited; also, the expression of several integrins is affected, inducing osteocyte apoptosis (Pesce Viglietti et al., 2016). This is not surprising given that integrins can link the cellular cytoskeletal network to the extracellular matrix (Geiger et al., 2001), and the detaching of osteocytes from the surrounding extracellular matrix was reported to induce anchorage-dependent cell death, anoikis (Gilmore, 2005).

Taking into account that osteocytes directly control bone remodeling, the modification of the activity of these cells by *B. abortus* infection could have an important contribution to bone damage observed during osteoarticular brucellosis.

## *Brucella* and Fibroblast-Like Synoviocytes

Fibroblast-like synoviocytes are mesenchymal cells that display many characteristics of fibroblasts (Bartok and Firestein, 2010). These cells have been recognized as central mediators of joint damage in inflammatory arthritis of either infectious or noninfectious origins (Inman and Payne, 2003; Bartok and Firestein, 2010).

Human synoviocytes infected with *B. abortus* secrete MMP-2, proinflammatory cytokines, RANKL, and chemokines that can promote the transmigration of monocytes and neutrophils and may mediate an increase in the synovium activation (Scian et al., 2011a). Several investigations have revealed the involvement of RANKL in bone destruction that occurs in rheumatoid arthritis and osteoarticular infectious diseases (Teitelbaum, 2006; Takayanagi, 2007). *Brucella* infection is not the exception as it has been demonstrated that synoviocytes infected with *B. abortus* induces osteoclastogenesis via RANKL, as proved using the natural inhibitor of osteoclastogenesis, OPG (Scian et al., 2013).

Besides their direct pathogenic role due to the production of proinflammatory mediators, *B. abortus* infection inhibits human synoviocyte apoptosis. Smooth *Brucella* species have developed several mechanisms to survive intracellularly, especially inside macrophages. It has been demonstrated that *B. abortus* infects and replicates in primary human synoviocytes (Scian et al., 2011a); this is in line with its capacity to replicate in other nonphagocytic cells such as osteoblasts, astrocytes, hepatocytes and hepatic stellate cells (Delpino et al., 2009, 2012; Garcia Samartino et al., 2010; Scian et al., 2011b). However, at variance with these cells, infection does not induce synoviocyte apoptosis. Moreover, it inhibits apoptosis induced by staurosporine and by culture supernatants from *B. abortus*-infected macrophages and monocytes. This inhibition occurs due to the up-regulation of anti-apoptotic factors such as cIAP-2, clusterin, livin, and P21/CIP/CDNK1A, and the reduction in the expression of pro-apoptotic proteins such as P-p53(S15) and the tumor necrosis factor (TNF) RI/TNFRSF1A (Scian et al., 2013). Thus, *B. abortus* could use these cells as an alternative replicative niche in joints. Accordingly, confocal imaging confirmed that as in macrophages, *B. abortus* replicates within calnexin-positive vacuoles in human primary synoviocytes (Scian et al., 2013). Therefore, interactions of *B: abortus* and synovial fibroblast may play an important role in the pathogenesis of osteoarticular diseases.

## Inmune Cells and Osteoarticular Brucellosis

### Interactions between Macrophages and *Brucella* in Osteoarticular Brucellosis

Upon infection with *B. abortus*, macrophages release inflammatory mediators that are able to induce the formation of osteoclasts from undifferentiated murine bone marrow cells (Delpino et al., 2012). In chronic inflammatory bone diseases such as rheumatoid arthritis, proinflammatory cytokines TNF-α, IL-1β, and IL-6, as well as RANKL, have been shown to be important in disease progression and bone loss (Nair et al., 1996; Merkel et al., 1999; Kotake et al., 2001; Haynes, 2004; Wei et al., 2005). TNF-α has been reported to stimulate osteoclastogenesis by a RANKL-independent mechanism (Azuma et al., 2000; Kobayashi et al., 2000). Macrophages infection with *Brucella* elicits the secretion of TNF-α, IL-1β, and IL-6 but not RANKL. Cytokine production by macrophages and concomitant osteoclastogenesis is not dependent on bacterial viability as both phenomena are induced by heat-killed *Brucella abortus*, suggesting that a structural component of *B. abortus* in responsible for such a response. *B. abortus* lipoproteins seem to be determinant as L-Omp19, a prototypical *B. abortus* lipoprotein (Giambartolomei et al., 2004), mimick the phenotypic and functional changes induced by *B. abortus* which lead to osteoclast activation. This phenomenon is caused by the lipid moiety of the protein as unlipidated Omp19 (U-Omp19) was unable to induce proinflamatory cytokine secretion and concomitant osteoclastogenesis. In addition, when using knockout mice, it was determined that MyD88 and TLR2 are both necessary to induce osteoclastogenesis by *B. abortus* and its lipoproteins as revealed by L-Omp19 stimulation (Delpino et al., 2012).

TNF-α is a potent inducer of bone resorption and it is the main proinflammatory cytokine involved in pathological conditions by activating mature osteoclasts (Thomson et al., 1987; Lerner and Ohlin, 1993; Kitazawa et al., 1994). With the use of TNFR1p55 knockout mice, it was determined that TNF-α signaling through TNFR1 appeared to be the main determinant of macrophage-elicited osteoclastogenesis induced by *B: abortus* and its lipoproteins. The major role of TNF-α in *B. abortus*-induced osteoclastogenesis was also determined in human cells using cytokine-neutralizing antibodies (Delpino et al., 2012). As osteoblasts and synoviocytes secrete MCP-1 in response to *B. abortus* infection (Scian et al., 2011a,b), monocytes can be attracted to the site of infection. In the *in vivo* situation attracted and resident-infected monocytes/ macrophage can respond to *Brucella* lipoproteins with the production of TNF-α, thus inducing osteoclastogenesis. In these conditions, proinflammatory cytokines from bone environment induce MCP-1 secretion by human and murine osteoblasts and human synoviocytes. Therefore, this interaction would create a pathological, vicious circle that exacerbates bone damage.

### Role of T Cells in *Brucella*-Induced Bone Loss

The interaction between T cells and osteoclasts is a critical issue in the field of bone infectious and non-infectious diseases (Takayanagi, 2007). Activated T cells tilt bone homeostasis and induce bone destruction under pathological conditions such as estrogen deficiency (Kong et al., 1999; Cenci et al., 2000; Roggia et al., 2001; Kawai et al., 2006) and inflammation (Kong et al., 1999; Kawai et al., 2006) as they become significant sources of RANKL (Kong et al., 1999), TNF-α (Cenci et al., 2000), and IL-17 (Sato et al., 2006). Although these cytokines could induce osteoclast differentiation (Takayanagi, 2010), most T-cell cytokines, as well as IFN-γ, IL-4 and IL-10 inhibit osteoclastogenesis (Takayanagi, 2010). Because infiltration of T cells into the bones and joints is a hallmark pathological finding of osteoarticular brucellosis (Madkour, 2001b; Giambartolomei et al., 2004), it is essential to address whether and how T cells are linked to enhanced osteoclastic bone resorption in this form of brucellosis.

In an *in vitro* model in which murine purified T cells are influenced by the inflammatory milieu elicited by *B. abortus*-infected macrophages, it was demonstrated that T cells may promote the generation of osteoclasts. The pre-activation of these T cells with anti-CD3 induces the secretion of IFN-γ. However, proinflammatory mediators from *B. abortus*-infected macrophages tilt this phenotype to T CD4+ cells to secrete RANKL and IL-17 (Giambartolomei et al., 2012). Although RANKL is the major cytokine that regulates osteoclast differentiation (Takayanagi, 2009), in this model based on the use of blocking anti-L-17 antibodies or osteoclast precursors from IL17R knockout mice it was revealed that IL-17 drives osteoclastogenesis induced by *Brucella*-activated T cells. In addition, IL-6 in the inflammatory milieu from *B. abortus-*infected macrophages is the cytokine that induces IL-17 secretion by T cells (Giambartolomei et al., 2012). IL-17 stimulates osteoclastogenesis indirectly through the induction of proinflammatory cytokines (TNF-α, IL-1β, and IL-6) by osteoclast precursors. Moreover, it was determined that, with the use of TNFRp55 knockout mice, TNF-α is the main proinflammatory cytokine involved in osteoclastogenesis in the context of T cells influenced by the inflammatory milieu elicited by *B. abortus*-infected macrophages.

It is well known that *Brucella* infection activates the immune system leading to a response that favors the differentiation of T cells toward a Th1 profile (Golding et al., 2001). This response, which involves mainly IFN-γ-producing T cells, is considered to be important in restraining infection (Zhan et al., 1993; Zhan and Cheers, 1995a,b). However, taking into account that there is increased recognition of plasticity within the T helper lineage (Nakayamada et al., 2012), IFN-γ-producing Th1 cells could turn into pathogenic Th17 cells under the influence of the local inflammatory milieu generated by *Brucella-*infected macrophages in the bone, leading to a pro-osteclastogenic T cell lineage.

### Influence of *Brucella*-Activated B Cells on Osteoclastogenesis

The primary function of B cells is the production of antimicrobial immunoglobulins against infecting pathogens. However, B cells may also contribute to the proinflammatory innate host response (Milner et al., 2005; Viau and Zouali, 2005). Activated B cells have long lifespan, and *B. abortus* infection activates B cells (Goenka et al., 2011). Also, B cells provide an infection niche for *B. abortus* (Goenka et al., 2012).

B cells have a close and multifaceted relationship with bone cells (Horowitz et al., 2010) in normal and in pathological conditions. Infiltrating B cells into bones and joints have been found (Young, 2008) in osteoarticular brucellosis. Taken together, these findings suggest that B cells could contribute to infection chronicity (Goenka et al., 2011). In this way, research on human subjects revealed that chronic brucellar lesions on bones and joints characteristically show, at the histological level, an inflammatory response with varying degrees of bone destruction and the presence of infiltrating lymphocytes (Madkour, 2001b). Studies performed *in vitro* with B cells purified from murine spleens have demonstrated that *B. abortus* infection induces the expression of MMP-9, RANKL, and proinflammatory cytokines. Besides the ability of proinflammatory cytokines and RANKL to induce osteoclastogenesis, OPG inhibits osteoclastogenesis induced by B cells indicating that RANKL is the main molecule involved in the induction of bone resorption through the increase in osteoclast differentiation (Pesce Viglietti et al., 2016).

## *In Vivo* Models of Osteoarticular Brucellosis

The slow progress made in defining the pathobiology of osteoarticular brucellosis has been hampered by the absence of a suitable animal model in which the variety of disease manifestations observed in humans can be reproduced after experimental infection. Despite not being natural hosts for *Brucella* species that cause diseases in humans, laboratory rodents do not either mimic the spectrum of clinical signs observed in humans. Therefore, although the challenges of clinical brucellosis are associated with its focal involvement, the pathophysiological manifestations in animal models of brucellosis remain poorly characterized.

However, until now, a few studies based on the use of murine models have reported the dynamics of *Brucella* infection in bone. In particular, knockout mice in the IFN-γ signaling pathway have been useful as *in vivo* models of osteoarticular brucellosis. In one of these models, the use of bioluminescent *B. melitensis* to infect intraperitoneally interferon regulatory factor-1 (IRF-1^-^/^-^) knockout mice enabled to identify acute infection in many tissues, even in the tail joint (Rajashekara et al., 2005). Similar results were obtained using *B. abortus*-infected IFN-γ knockout mice (Skyberg et al., 2012). Identification of joint localization may provide a model to understand bone pathogenesis of chronic brucellosis in humans (Rajashekara et al., 2005). Wild type Balb/c mice rarely develop spontaneous inflammation in synovial joints (Adarichev et al., 2008; Farkas et al., 2009). However, Balb/c mice infected for 26 weeks with bioluminescent *B. melitensis* exhibited multiple sites of the axial skeletal involvement with inflammatory and non-inflammatory features (Magnani et al., 2013). In these mice, brucellosis-induced arthritis is a progressive disease with postponed onset. In contrast, IRF-1 or IFN-γ knockout mice (Rajashekara et al., 2005; Skyberg et al., 2012; Lacey et al., 2016) develop arthritis much earlier, when *Brucella* is found in high concentrations in the body during the acute stage of the disease. In these conditions, inflammation and presence of bacteria was limited to paw joints and tails (Rajashekara et al., 2005; Skyberg et al., 2012; Lacey et al., 2016).

Despite the limitations of using animal models to study *Brucella* infection, the extensive bacterial dissemination in the murine host in the conditions indicated above raises novel possibilities for the use of these models. Osteoarticular complications are particularly common in *Brucella*-infected humans, and the mouse model of brucellosis is particularly useful for the use of guided imaging techniques to identify infectious osteoarticular foci. Interestingly, inflammation and *Brucella* foci were independent of the infection route, suggesting that the osteoarticular site is a preferred location for bacterial persistence in the host and the most inflammation-susceptible structure (Skyberg et al., 2012).

The intra-articular knee injection of heat-killed *Brucella* further suggests that joint infection can induce a pro-inflammatory environment, with the induction of osteoclastogenesis (Scian et al., 2011a; Delpino et al., 2012). In addition, this approach allows corroborating *in vitro* findings such as the role of TNF-α, MyD88 and TLR-2 in *Brucella-* induced osteoclatogenesis (Delpino et al., 2012), and the role of T cells in *Brucella-*induced osteoclastogenesis (Giambartolomei et al., 2012). Further exploration of this model should allow us to determine the relevant parallels to human clinical outcomes.

## Concluding Remarks

**FIGURE 1:**
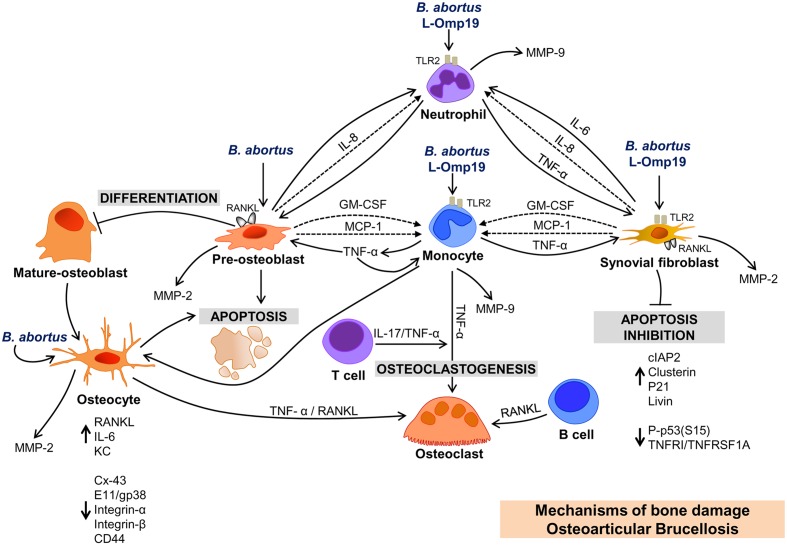
**Bone damage induced by soluble mediators secreted by resident and infiltrating cells upon infection *with Brucella abortus*.** *OSTEOBLAST: B. abortus* infection of osteoblast induces RANKL, MMP-2, GM-CSF, and chemokines (IL-8 and MCP-1) expression; induces apoptosis, and inhibits osteoblast differentiation. Chemokines attract monocytes and neutrophils to the site of infection, and these cells secrete MMP-9. TNF-α secreted by *B. abortus-*infected monocytes inhibits differentiation and induces apoptosis and RANKL expression in osteoblasts. It is the main cytokine involved in the secretion of MMP-9 by monocytes. *OSTEOCYTE: B. abortus* infection induces MMP-2, RANKL proinflamatory cytokines, and KC secretion by osteocytes. TNF-α and RANKL from *B. abortus-*infected osteocytes induce osteoclastogenesis. *B. abortus* infection inhibits the expression of Cx43, but does not modify integrins expression. In contrast, supernatants from *B. abortus* infected macrophages inhibit Cx43 and integrins inducing osteocyte apoptosis. *SYNOVIAL FIBROBLAST: B. abortus* infection induces MMP-2 and RANKL expression, and inhibits synoviocyte apoptosis through the upregulation of anti-apoptotic factors (cIAP-2, clusterin, livin, and P21/CIP/CDNK1A) and the reduction in the expression of proteins involved in apoptosis (P-p53(S15) and TNFRI/TNFRSF1A). *IMMUNE CELLS AND OSTEOARTICULAR BRUCELLOSIS*. Supernatants from *B. abortus*-infected macrophages induce osteoclastogenesis via TNF-α induction. T cells secrete IL-17 in response to supernatants from *B. abortus*-infected macrophages, which induce osteoclastogenesis via TNF-α secreted by osteoclast precursors. *B. abortus*-infected B cells secrete RANKL that induce osteoclastogenesis. L-OMP19. *Brucella* lipoproteins mimic responses mediated by *B. abortus* infection in neutrophils, monocytes and synovial fibroblasts and these responses require TLR2.

Osteoarticular brucellosis is the most common localization of active brucellosis, and bone loss has been reported consistently in its three most frequent forms of osteoarticular involvement (Young, 1989; Madkour, 2001a,b; Aydin et al., 2005; Colmenero et al., 2008). Although the clinical and imaging aspects of osteoarticular brucellosis have been widely described (Madkour, 2001a,b), the pathogenic mechanisms of bone loss caused by *Brucella* have only been partially elucidated at the molecular and cellular levels over the past ten years (**Figure 1**). The findings presented in this review try to answer key questions about the inflammatory mediators involved in osteoarticular damage caused by *Brucella* and provides an initial background for studying in more detail the role of local and infiltrating cells in this localization of the disease. As the infection inhibits apoptosis -or cell death- in some osteoarticular cells, it could be possible that these cells act as reservoir of the bacteria allowing the evolution of the disease to chronicity. This could also indicate which cell type may be chosen for therapeutic targeting.

This knowledge could lead to the discovery of new therapeutic treatments that could be co-administered with antibiotics to improve the patient’s response to infection and reduce bone damage.

## Author Contributions

GG helped to draft and to correct the manuscript. PA performed the figure and critical reading of the manuscript. MD drafted the manuscript.

## Conflict of Interest Statement

The authors declare that the research was conducted in the absence of any commercial or financial relationships that could be construed as a potential conflict of interest.
